# Association Between Impaired Vα7.2+CD161++CD8+ (MAIT) and Vα7.2+CD161-CD8+ T-Cell Populations and Gut Dysbiosis in Chronically HIV- and/or HCV-Infected Patients

**DOI:** 10.3389/fmicb.2019.01972

**Published:** 2019-08-28

**Authors:** Esther Merlini, Maddalena Cerrone, Bonnie van Wilgenburg, Leo Swadling, E. Stefania Cannizzo, Antonella d’Arminio Monforte, Paul Klenerman, Giulia Marchetti

**Affiliations:** ^1^Department of Health Sciences, Clinic of Infectious Diseases, ASST Santi Paolo e Carlo, University of Milan, Milan, Italy; ^2^Chelsea and Westminster Hospital NHS Foundation Trust, London, United Kingdom; ^3^Peter Medawar Building for Pathogen Research, University of Oxford, Oxford, United Kingdom

**Keywords:** mucosal-associated invariant T-cells, HIV infection, HCV infection, microbial translocation, gut microbiota, dysbiosis

## Abstract

Both HIV and HCV infections feature increased microbial translocation (MT) and gut dysbiosis that affect immune homeostasis and disease outcome. Given their commitment to antimicrobial mucosal immunity, we investigated mucosal-associated invariant T (MAIT) cells and Vα7.2+CD161- T-cell frequency/function and their possible associations with MT and gut dysbiosis, in chronic HIV and/or HCV infections. We enrolled 56 virally infected (VI) patients (pts): 13 HIV+ on suppressive cART (HIV-RNA < 40cp/ml), 13 HCV+ naive to DAA (direct-acting antiviral) anti-HCV agents; 30 HCV+/HIV+ on suppressive cART and naive to anti-HCV. 13 age-matched healthy controls (HC) were enrolled. For Vα7.2+CD161++ and Vα7.2+CD161-CD8+ T cells we assessed: activation (CD69), exhaustion (PD1/CD39), and cytolytic activity (granzymeB/perforin). Following PMA/ionomycin and *Escherichia coli* stimulation we measured intracellular IL17/TNFα/IFNγ. Markers of microbial translocation (Plasma LPS, 16S rDNA, EndoCAb and I-FABP) were quantified. In 5 patients per group we assessed stool microbiota composition by 16S targeted metagenomics sequencing (alpha/beta diversity, relative abundance). Compared to controls, virally infected pts displayed significantly lower circulating Vα7.2+CD161++CD8+ MAIT cells (*p* = 0.001), yet expressed higher perforin (*p* = 0.004) and granzyme B (*p* = 0.002) on CD8+ MAIT cells. Upon *E. coli* stimulation, the residual MAIT cells are less functional particularly those from HIV+/HCV+ patients. Conversely, in virally infected pts, Vα7.2+CD161-CD8+ cells were comparable in frequency, highly activated/exhausted (CD69+: *p* = 0.002; PD-1+: *p* = 0.030) and with cytolytic potential (perforin+: *p* < 0.0001), yet were poorly responsive to *ex vivo* stimulation. A profound gut dysbiosis characterized virally infected pts, especially HCV+/HIV+ co-infected patients, delineating a *Firmicutes*-poor/*Bacteroidetes-*rich microbiota, with significant associations with MAIT cell frequency/function. Irrespective of mono/dual infection, HIV+ and HCV+ patients display depleted, yet activated/cytolytic MAIT cells with reduced *ex vivo* function, suggesting an impoverished pool, possibly due to continuous bacterial challenge. The MAIT cell ability to respond to bacterial stimulation correlates with the presence of *Firmicutes* and *Bacteroidetes*, possibly suggesting an association between gut dysbiosis and MAIT cell function and posing viral-mediated dysbiosis as a potential key player in the hampered anti-bacterial MAIT ability.

## Introduction

Mucosal-associated invariant T (MAIT) cells are unconventional T lymphocytes that are relatively abundant in humans, representing 1–10% of circulating T-cells and which are further enriched in mucosal tissues, such as gut lamina propria and liver (Dusseaux et al., 2011; Tang et al., 2013). MAIT cells are characterized by the expression of CD161 and an invariant T cell receptor α segment (Vα7.2) (Dias et al., 2017) that recognizes and is activated by small vitamin metabolites produced by microbes and presented by the major histocompatibility complex class I-related molecule MR1 (Le Bourhis et al., 2010, 2011; Kjer-Nielsen et al., 2012; Gapin, 2014).

Once activated, MAIT cells respond rapidly by producing cytokines and cytolytic products. Indeed, upon TCR-dependent or TCR-independent activation [mediated by IL-12/IL-18 (Ussher et al., 2014; Sattler et al., 2015)], MAITs produce interferon-γ (IFN-γ), tumor necrosis factor-α (TNF-α), IL-17, cytolytic products (perforin and granzymes) and degranulate (exposing CD107a to the cell surface) (Dusseaux et al., 2011; Le Bourhis et al., 2013; Kurioka et al., 2015).

Increasing evidence suggests that MAIT cells play a protective role in anti-bacterial immunity at mucosal interfaces (Le Bourhis et al., 2010, 2011; Salou et al., 2017; Wong et al., 2017). While the exact mechanisms by which MAITs exert their function are not fully assessed, it has been shown that they are depleted in many bacterial infections including active tuberculosis (Jiang et al., 2014), vibrio cholera infection (Leung et al., 2014) and septic shock (Grimaldi et al., 2014), possibly supporting a role of MAITs in controlling bacterial threats.

Unexpectedly, despite the fact that viruses lack the metabolic pathways to synthesize riboflavin and therefore do not directly activate MAIT cells via MR1, several authors have reported a massive and irreversible loss of MAIT cells in peripheral blood, as well as in mucosal tissues of HIV and/or HCV chronically infected individuals (Leeansyah et al., 2013; Fernandez et al., 2015; Barathan et al., 2016; Eberhard et al., 2016; Hengst et al., 2016; Saeidi et al., 2016; Spaan et al., 2016; Bolte et al., 2017). The nature of this impairment is not clear. While Cosgrove et al. (2013) reported that MAIT cell depletion was due to activation-induced cell death from over-stimulation secondary to microbial translocation, Leeansyah et al. (2013) suggested that continuous exposure to bacterial products in HIV disease may lead to MAIT cell exhaustion and loss, with concomitant expansion of the CD161neg population originated from the MAIT cells.

In the last few years, the understanding of the interactions between gut mucosal immunity and microbiota has significantly broadened (Powell and MacDonald, 2017; Shi et al., 2017). Intestinal microbiota shapes host immunity and contribute to the maintenance of intestinal homeostasis and to the inhibition of excessive inflammation (Lee and Mazmanian, 2010; McDermott and Huffnagle, 2014; Sun et al., 2015). Indeed, an impaired interaction between intestinal microbiota and the mucosal immune system is associated with the pathogenesis of several inflammatory diseases, such as inflammatory bowel disease, rheumatoid arthritis, systemic lupus erythematosus and ankylosing spondylitis (Mazmanian et al., 2008; Hevia et al., 2014; Wang et al., 2014; Costello et al., 2015; Wright et al., 2015; Zhang et al., 2015). Similarly, during chronic HCV and HIV infections a marked dysbiosis has been described, which is not reverted by anti-HCV or anti-HIV treatments (Bajaj et al., 2016; Williams et al., 2016), highlighting the need to explore the function of microbiota in such diseases.

Given that MAIT cells are predominantly present in the gastrointestinal tract and in the liver, where they exert their antimicrobial function and help fight off bacterial infection by responding to infected cells and producing cytokines, it is plausible to hypothesize a role for MAIT cells in maintaining intestinal homeostasis, through the wide reactivity toward several microbial species, including commensal organisms (Gaardbo et al., 2015; Johansson et al., 2016). Thus, in a cohort of chronically infected HIV+ and/or HCV+ patients, we aimed to investigate MAIT cells and Vα7.2+CD161- T-cell phenotype and function and the associations with microbial translocation and gut dysbiosis.

## Materials and Methods

### Study Population

HIV+, HCV+, HCV+/HIV+ infected patients (age >18 years old) were enrolled from January 2009 to December 2016 at the Clinic of Infectious Diseases, University of Milan – ASST Santi Paolo e Carlo, Milan, after providing written, informed consent (approved by the Institutional Review Board at the ASST Santi Paolo e Carlo, Milan, Italy) in accordance with the Declaration of Helsinki. Inclusion criteria were: (i) HIV infection: patients on virally suppressive cART (HIV-RNA < 40 cp/ml), and any current CD4 level; (ii) chronic HCV infection: patients naïve to direct acting antiviral (DAA)-based anti-HCV therapy, and any detectable HCV-RNA. Thirteen HIV-negative age-matched healthy subjects were enrolled as controls (HC). All patients underwent blood sampling, while in a subgroup of 5 subjects per group we also collected stool samples for metagenomic analyses. For the metagenomic analyses, patients were randomly selected, blood and stool were collected the same day and processed for DNA extraction within 4 months. Liver fibrosis was assessed using liver elastography (FibroScan^®^, Echosens, Paris, France) and Fib-4 score and categorized as absent to moderate (Metavir-score F0-F2) or severe to liver cirrhosis (Metavir-score F3-F4, Fib-4 score ≥ 3.25). No subject with decompensated liver disease was included in the study.

### T-Cell Immune Phenotypes

Lymphocyte surface phenotypes were evaluated by flow cytometry on cryopreserved PBMCs. Cell viability was assessed by live/dead (Invitrogen): only samples with viability greater than 90% were used for the experiments. Surface antibodies were incubated at 4°C for 20 min. For the evaluation of the intracellular markers a fixation/permeabilization step was required. The following fluorochrome-labeled anti-human antibodies were used: CD3 APC-H7/PE-Cy7 (BD Biosciences), CD3 PB (Biolegend), CD8 PE-Cy5 (BD Biosciences), CD8 PerCP.Cy5.5/PerCP (Biolegend), CD161 APC (BD Biosciences), CD161 PE (Miltenyi Biotech), CD4 VioGreen (Miltenyi Biotech), CD39 FITC (BD Biosciences), PD-1 PE/PB (BD Biosciences), CD69 FITC (BD Biosciences), Granzyme B PE (BD Biosciences), Granzyme B APC (Invitrogen, PERFORIN A488 (BD Biosciences), Perforin PB (Invitrogen), LIVE/DEAD (Thermo Fisher Scientific), LIVE/DEAD (Invitrogen), IL18R PE (Biolegend), IL-18Ra APC (eBioscience), TCR Vα7.2 PE-Cy7/APC/FITC(Biolegend). MAIT cells were defined as Vα7.2+CD161++CD3+ for total MAIT cell population and Vα7.2+CD161++CD8+. We evaluated MAIT cell activation (CD69), exhaustion (CD39/PD-1), IL18R expression and cytolytic activity (Granzyme B/perforin). For intracellular staining PBMCs were fixed with PFA 1% for 30 min, then washed and permeabilized with saponin 0.2% for 30 min at room temperature (RT). To verify that different labelled-antibodies gave similar results, we thawed the same cell batch of PBMCs and performed titration curves of the antibodies, evaluating the stain index, the height and the width of the peak and the resolution. Cells were acquired using a FACSVerse flow cytometer (Becton Dickinson) or MACSQuant (Miltenyi Biotech). Five patients were used to standardize the acquisition and analysis of the MAIT cells on two different cytometers. In particular, the same batch of PBMCs was thawed, stained and split in 2 tubes, each tube was run in one cytometer alone. Results were then compared. The axes were set based on positive versus negative.

### *In vitro* Infection and Cell Activation

*Escherichia coli* (DH5α, Invitrogen) was cultured overnight at 37°C in SOC broth. Bacteria were washed once in PBS and fixed in 2% paraformaldehyde for 20 min, then washed extensively before counting by Flow Cytometry (FACSVerse, BD Biosciences) and added to the THP1 (a human monocytic cell line derived from an acute monocytic leukemia patient) in a bacteria/cell ratio of 100:1 for 24 h. PBMCs were cultured for 1 h in round-bottom 96-well plates in the presence of *E. coli*-activated THP1 (1:2) or PMA (250 ng/μl) plus ionomycin (1 μg/μl) as positive control. Brefeldin A (10 μg/ml) was added and cells were incubated for further 4 h. Cells were harvested and stained as previously described. For the intracellular quantification of cytokine production, the following antibodies were used: IL-17 PE (Miltenyi Biotech), IFNγ PE and TNFα FITC (BD Biosciences).

The assay setup for both PMA/iono and bacterial stimulation was optimized on 2 HIV– and 1 HIV+ subjects. During each round of experiments, PBMCs from the same batch of the same HIV– subject were included. This allowed us to verify that the culture conditions were reliable and that the stimuli were working as expected.

### Microbial Translocation (MT) and Gut Damage Markers

Endotoxin Core Antibodies (EndoCab) and Intestinal Fatty Acid Binding Protein (I-FABP) were measured by ELISA (R&D systems), in accordance with the manufacturer’s instructions. Circulating lipopolysaccharide (LPS) was assessed using the LAL test (Lonza), as per the manufacturer’s instructions. Samples were diluted 1:150 and preheated at 95°C for 10 min.

### 16S rDNA Quantification and Metagenomic Sequencing of Blood and Fecal Samples

Total DNA was extracted as previously described (Paisse et al., 2016). The V3-V4 hypervariable regions of the 16S rDNA were amplified and quantified by qPCR, sequenced with MiSeq technology, and clustered into operational taxonomic units (OTUs) before taxonomic assignment as described (Garidou et al., 2015; Lelouvier et al., 2016; Paisse et al., 2016).

The total 16S rDNA present in the samples was measured by qPCR in triplicate and normalized using a plasmid-based standard scale (Vaiomer SAS, Labége, France). The targeted metagenomic sequences from fecal and plasma microbiota were analyzed using the bioinformatics pipeline established by Vaiomer from the FROGS guidelines. Briefly, after demultiplexing of the barcoded Illumina paired reads, single read sequences were cleaned and paired for each sample independently into longer fragments. After quality-filtering and alignment against a 16S reference database, a clustering into OTU with a 97% identity threshold and a taxonomic assignment were performed in order to determine community profiles. The list of possible species for each unique sequence was further investigated using the BLASTN program from NCBI Blast against the NCBI 16S Microbial database. Only the BLASTN hits covering the full query sequence length with an overall sequence identity of 97% or more were considered as possible species. DNA from plasma (240 mL) was extracted using a DNA isolation kit (NucleoSpin Plasma XS, Macherey-Nagel). All DNA extracts were stored at –80°C until further processing.

### Bioinformatics Analyses

Reads obtained from the MiSeq sequencing system have been processed using Vaiomer bioinformatics pipeline. Based on the results, graphical representations were made of the relative proportion of taxa for each taxonomic level (phylum, class, order, family, genus, and species) present in individual study samples. Taxa are identified by name in the plot for abundance >1%. Taxa are merged into the “Other” category only if it exists in any sample with abundance greater than 0.01%. Taxa are merged into the “Multi-affiliation” category when they can correspond to two or more different taxa. Alpha diversity (α-diversity) represents the mean of species diversity per sample in each group/class. Diversity analysis is presented for (1) observed, (2) Chao1, (3) Shannon, (4) Simpson, and (5) inverse Simpson. Finally, the output matrix containing the relative abundance of OTUs per sample was processed with the linear discriminant analysis effect size (LEfSe) algorithm (Segata et al., 2011) using an alpha cut-off of 0.05 and an effect size cut-off of 2.0.

### Statistical Analyses

Continuous variables were expressed as median and interquartile range (IQR), whereas categorical variables were expressed as absolute numbers and percentages. The different groups of patients and the different time points were compared using Chi-squared or Fisher’s exact test, Kruskal–Wallis or Wilcoxon matched pairs test as appropriate and the correlations among variables were tested by Spearman Rank correlation. Dunn’s multiple comparison test was performed as appropriate: a line below the *p*-value showing which groups are being compared and resulted significant has been added in each graph. *p*-Values < 0.05 were considered statistically significant. Data were analyzed with GraphPad 6.2 Prism (GraphPad Software Inc.).

## Results

### Study Population

A total of 13 HIV+ mono-infected, 13 HCV+ mono-infected, 30 HCV+/HIV+ co-infected patients and 13 age-matched HIV- and HCV-uninfected healthy controls (HC) were enrolled in the study. Patients’ epidemiological and clinical characteristics are presented in Table 1. Virally infected patients were preferentially men (*p* = 0.031, Table 1), with a higher proportion of men who have sex with men (MSM) and intravenous drug users (IVDU) (*p* < 0.0001). With regard to HIV-related features, HIV+ and HCV+/HIV+ patients were comparable in terms of CD4 nadir and % of CD4 count at time of analysis, duration of infection and suppressive cART (Table 1). With regard to HCV infection, HCV+/HIV+ patients showed significantly higher levels of circulating HCV-RNA (*p* = 0.007, Table 1) compared to HCV+, yet similar duration of infection, liver fibrosis, as assessed by ultrasound transient elastography and HCV genotype distribution (Table 1). As expected, HCV+ infected patients (mono-infected and HIV+ co-infected) showed higher serum transaminases compared to both healthy controls and HIV+ individuals (*p* < 0.0001, Table 1).

**TABLE 1:** Epidemiological, clinical and immunological characteristics of the study groups.

	**HIV (*n* = 13)**	**HCV (*n* = 13)**	**HCV/HIV (*n* = 30)**	**HC (*n* = 13)**	***p***
Age, years (IQR)^∗^	45 (36–52)	49 (45–56.5)	48.5 (41–53)	42 (32.5–52)	0.175^*a*^
Sex, Male (%)°	9 (69)	11 (85)	26 (87)	6 (46)	**0.031^a^**
Risk Factors, (%)° Heterosex Homo/Bisex IDU Unknown/others	7 (54) 6 (46) 0 0	0 0 6 (46) 7 (54)	1 (3) 9 (30) 20 (67) 0	11 (85) 2 (15) 0 0	**<0.0001^a^**
AST, U/L (IQR) ^∗^	26 (19.5–34.5)	63 (47–95)	57.5 (34–80)	24.5 (20.5–29.5)	**<0.0001^a^**
ALT, U/L (IQR) ^∗^	67 (45.5–132.5)	80.5 (41.75–131.8)	28 (22–35)	**<0.0001^a^**	
**HIV-related parameters**					
Current CD4, cell/mmc (IQR) ^∗^	748 (594–884)	n/a	518 (398–775)	n/a	**0.033^b^**
Current CD4,% (IQR) ^∗^	33 (28–35)	n/a	28.5 (21–35)	n/a	0.112^b^
Nadir CD4, cell/μl (IQR) ^∗^	220 (84–326)	n/a	158 (57–289)	n/a	0.344^b^
CD4/CD8 Ratio (IQR) ^∗^	0.78 (0.58–0.91)	n/a	0.61 (0.37–0.83)	n/a	0.128^b^
Time since 1° HIV diagnosis, years (IQR) ^∗^	9 (6.5–16.5)	n/a	19 (10–26)	n/a	0.131^b^
cART duration, years (IQR) ^∗^	9 (7–14)	n/a	9.5 (5–17)	n/a	1^b^
HIV-RNA, Log cp/ml (IQR) ^∗^	1.59 (1.59–1.59)	n/a	1.59 (1.59–1.59)	n/a	0.197^b^
Patients with undetectable HIV-RNA, *n* (%)°	13 (100)	n/a	30 (100)	n/a	1^b^
**HCV-related parameters**					
Time since 1° HCV diagnosis, years (IQR) ^∗^	n/a	13 (6–22)	19 (10–26)	n/a	0.222^c^
HCV-RNA, cp/ml (IQR) ^∗^	n/a	256536 (42730–425654)	2178000 (262522–4089000)	n/a	**0.007^c^**
HCV Genotype, (%)° 1a 1b 3 4	n/a	4 (31) 8 (62) 1 (7) 0	15 (50) 8 (27) 3 (10) 4 (13)	n/a	0.138^c^
Liver Fibrosis, (%)° F0-F2 F3-F4	n/a	8 (61) 5 (39)	25 (83) 5 (17)	n/a	0.139^c^
HBsAg, yes (%)°	n/a	0	2 (6)	n/a	1^c^
PLT, n (IQR) ^∗^	n/a	130000 (73000–201500)	184000 (154500–210750)	n/a	0.076^c^
Peginterferon/ribavirin-experienced, yes (%)	n/a	7 (54)	7 (23)	n/a	0.07^c^

*^∗^Data are presented as median (Interquartile range; IQR), statistical analyses: *t*-test or ANOVA as appropriate. °data are presented as absolute numbers (%), statistical analyses: Fisher’s or Chi-squared test as appropriate. ^*a*^*p*-values among 4 groups; ^*b*^*p*-values among HIV+ and HCV+/HIV+ groups; ^*c*^*p*-values among HCV+ and HCV+/HIV+ groups. IDU: intravenous drug user; AST: Aspartate Aminotransferase; ALT: Alanine Transaminase; cART: combination antiretroviral therapy; PLT: platelet count; n/a: not applicable. The bold values indicate the statistical significant values.*

### Virally Infected Patients Display Lower Circulating Vα7.2+CD161++CD8+, With Higher Cytolysis Markers

**FIGURE 1:**
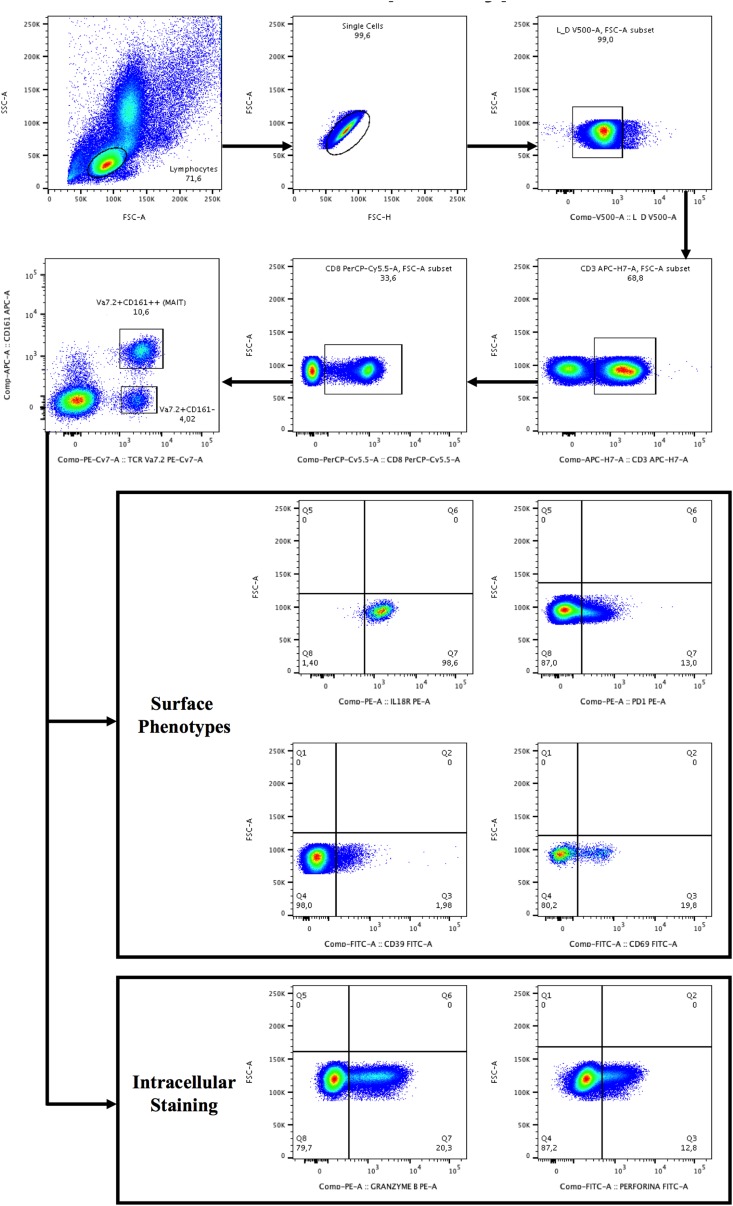
Gating strategy for the identification of Vα7.2+CD161++CD8+ and Vα7.2+CD161-CD8+ T-cell subset and surface/intracellular phenotypes. Lymphocytes were gated from forward (FSC) and side scatters (SSC), doublets were removed and live cells were selected, segregated for CD3+ T-cells and subsequently for CD8+ T-cells. CD8+ T+cells were categorized as CD161- or CD161++ (bright), followed by the selection of TCR Vα7.2. The gates were set up based on positive versus negative peak. Depicted are representative plots from representative subject PBMCs.

**FIGURE 2:**
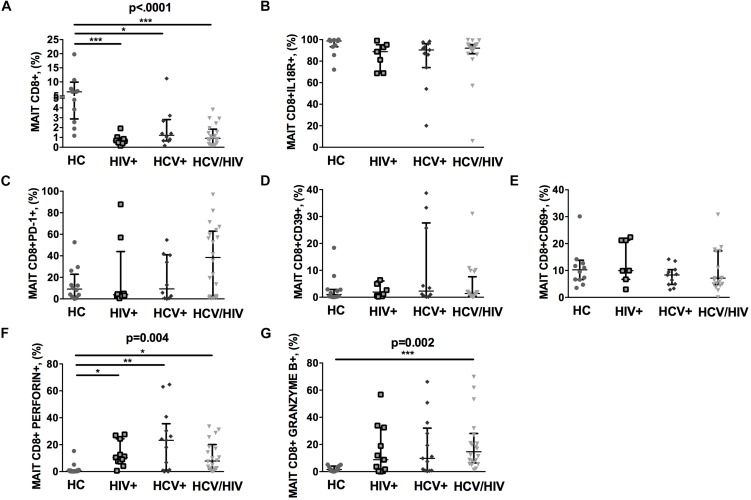
Frequency and phenotypes of Vα7.2+CD161++CD8+ T cells. The lines indicate the significant comparison between two groups. ^∗^ indicates the *p*-value for each pair of groups: ^∗^ *p* < 0.05, ^∗∗^ *p* < 0.01, ^∗∗∗^ *p* < 0.001. **(A)** Compared to healthy individuals (HC), virally infected patients displayed a significant lower proportion of peripheral MAIT cells (HC: 6.47%, HIV+: 0.65%, HCV+: 1.22%, HCV+/HIV+: 0.91%, *p* < 0.0001). **(B)** Virally infected individuals showed similar IL18R-expressing MAIT cells (HC: 98.70%, HIV+: 88.8%, HCV+: 90.4%, HCV+/HIV+: 92%, *p* = 0.061). **(C)** Similar levels of PD-1-expressing MAIT cells (HC: 9.15%, HIV+: 3.84%, HCV+: 9.28%, HCV+/HIV+: 38.45%, *p* = 0.236), **(D)** CD39-expressing MAIT cells (HC: 1.04%, HIV+: 1.91%, HCV+: 2.30%, HCV+/HIV+ 1.38%, *p* = 0.774), and **(E)** CD69-expressing MAIT cells (HC: 10.25%, HIV+: 9.96%, HCV+: 8.33%, HCV+/HIV+: 7.15%, *p* = 0.414) were observed among study groups. **(F)** Compared to the uninfected controls, virally infected patients featured higher perforin-producing (HC: 0.59%, HIV+: 10.5%, HCV+: 23.2%, HCV+/HIV+: 7.86%, *p* = 0.004) and **(G)** granzyme B-producing MAITs (HC: 1.53%, HIV+: 8.78%, HCV+: 9.79%, HCV+/HIV+: 14.6%, *p* = 0.002).

The CD3+ MAIT cell population comprises CD4+ and CD4/CD8 double negative (DN) subsets, along with the CD8+ subpopulation. Given that CD8+ MAIT cells represent the majority of circulating MAIT, we decided to focus our attention on this specific subset. We first identified circulating Vα7.2+CD161++CD8+ (MAIT) by flow cytometry (gating strategy shown in Figure 1). Compared to healthy individuals, virally infected patients displayed a significantly lower proportion of peripheral MAIT cells (*p* < 0.0001; Figure 2A), with no differences in IL18R-expressing Vα7.2+CD161++CD8+ (*p* = 0.061; Figure 2B). No differences in PD-1- (*p* = 0.236; Figure 2C), CD39-expressing Vα7.2+CD161++CD8+ (*p* = 0.774; Figure 2D) and activated CD69+Vα7.2+CD161++CD8+ (*p* = 0.414; Figure 2E) were shown in virally infected subjects when compared to HC. Conversely, virally infected patients displayed significantly higher perforin+ (*p* = 0.004; Figure 2F) and granzyme B+ MAIT cells (*p* = 0.002; Figure 2G), with no differences between the three groups of infected patients. Comparable MAIT frequency and phenotypes were shown in patients stratified according to liver fibrosis (F0–F2 vs. F3–F4) (data not shown). Similar data were shown with regard to total CD3+ MAIT cells: virally infected patients were confirmed to display a lower Vα7.2+CD161++CD3+ frequency (*p* = 0.003) with a similar proportion of activated/exhausted MAIT, yet higher cytolytic activity (granzyme B: *p* = 0.007) versus healthy controls (Supplementary Figures S1a–g). Furthermore, virally infected patients presented lower frequency of CD4/CD8 double negative MAIT (HIV–: 6.14% vs. HIV+: 0.36% vs. HCV+: 0.22% vs. HCV+/HIV+ 1.56%; *p* < 0.0001), yet higher level of PD-1-expressing (HIV–: 5.53% vs. HIV+: 4.07% vs. HCV+: 5.52% vs. HCV+/HIV+ 42.1%; *p* = 0.010), Granzyme B-expressing (HIV–: 0.19% vs. HIV+: 15.4% vs. HCV+: 19.3% vs. HCV+/HIV+ 3.7%; *p* = 0.003) and Perforin-expressing CD4/CD8 double negative MAIT (HIV–: 0.37% vs. HIV+: 1.12% vs. HCV+: 2.3% vs. HCV+/HIV+ 27.8%; *p* = 0.0027).

### Functional Analysis of MAIT Cells Reveals Reduced Responsiveness in HCV+/HIV+ Co-infected Group

**FIGURE 3:**
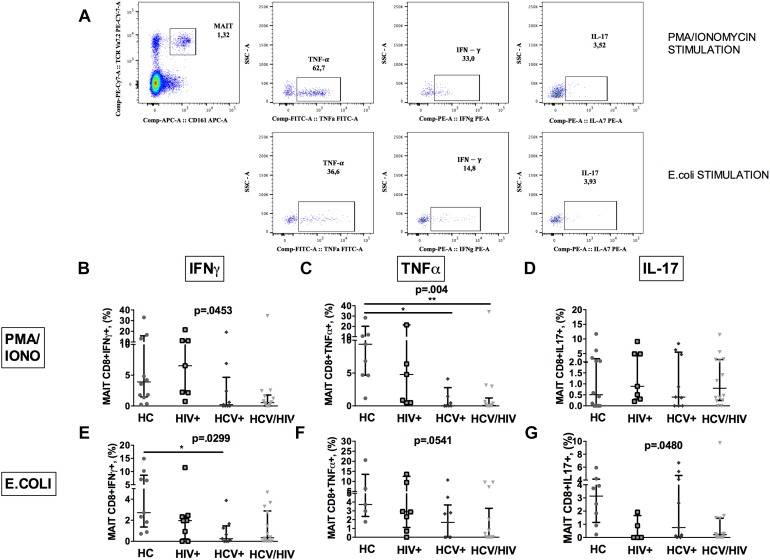
Vα7.2+CD161++CD8+ cytokine production following *ex vivo* stimulation. The lines indicate the significant comparison between two groups. ^∗^ indicates the *p*-value for each pair of groups: ^∗^ *p* < 0.05, ^∗∗^ *p* < 0.01, ^∗∗∗^ *p* < 0.001. **(A)** Flow cytometry gating strategy for cytokine production. **(B)** Stimulation with PMA/ionomycin resulted in significantly lower production of IFNγ (HC: 3.57%, HIV+: 4.39%, HCV+: 0.22%, HCV+/HIV+: 0.54%, *p* = 0.045) in virally infected, particularly co-infected individuals, compared to HC. **(C)** Co-infected individuals showed the lowest TNFα release after PMA/ionomycin (HC: 8.11%, HIV+: 4.39%, HCV+: 0.02%, HCV+/HIV+: 0.07%, *p* = 0.004). **(D)** Similar production of IL-17 between the study groups following PMA/ionomycin exposure (HC: 0.43%, HIV+: 0.70%, HCV+: 0.40%, HCV+/HIV+: 0.80%, *p* = 0.883). **(E)** Following *E. coli* stimulation, MAITs of infected patients produced inadequate quantity of IFNγ (HC: 2.72%, HIV+: 1.96%, HCV+: 0.25%, HCV+/HIV+: 0.33%, *p* = 0.025). **(F)** Virally infected patients showed lower TNFα production upon bacterial challenge (HC: 3.73%; HIV+: 0.17%, HCV+: 1.69%, HCV+/HIV+: 0.01%, *p* = 0.054) **(G)** *E. coli* exposure resulted in lower IL-17 production in virally infected patients vs. HC (HC: 3.14%, HIV+: 0%, HCV+: 0.75%, HCV+/HIV+: 0.21%, *p* = 0.048).

To further characterize MAIT cells during chronic viral infections, we sought to investigate their ability to produce cytokines following *in vitro* challenge (Figure 3A shows the gating strategy).

PMA/ionomycin stimulation resulted in significantly lower production of IFNγ (*p* = 0.0453; Figure 3B) and TNFα (*p* = 0.004; Figure 3C), but not of IL-17 (*p* = 0.883; Figure 3D) in virally infected, particularly HCV+/HIV+ co-infected individuals, compared to controls.

Having shown dysfunctional MAIT intracellular cytokine production and given the role of microbial translocation in supporting immune inflammation/activation (Brenchley et al., 2006), we next investigated MAIT response to *ex vivo* bacterial challenge. Upon *E. coli* stimulation, a lower percentage of MAIT cells from virally infected patients produced IFNγ (*p* = 0.0299; Figure 3E), TNFα (*p* = 0.0541; Figure 3F) and IL-17 (*p* = 0.048; Figure 3G) when compared to uninfected controls, which overall suggests a defective anti-bacterial MAIT effector function in chronically virally infected patients.

Similarly, total CD3+ MAIT cells of virally infected subjects showed lower production of cytokines following both PMA/ionomycin (IFNγ: *p* = 0.078; TNFα: *p* = 0.030) and *E. coli* exposure (IFNγ: *p* = 0.028; TNFα: *p* = 0.048) as compared to healthy individuals (Supplementary Figures S1h–m). CD4/CD8 double negative MAIT cell subset confirmed the same trend (data not shown).

### Virally Infected Patients Show Lower Plasma EndoCAb, Yet Similar LPS, 16S rDNA and I-FABP Levels

Given the well-recognized role of microbial translocation (MT) in sustaining chronic immune activation and disease progression in HIV infection (Brenchley et al., 2006; Paiardini et al., 2008; Estes et al., 2010; Marchetti et al., 2011, 2013), we explored the possible association between MAIT impairment and markers of damaged intestinal integrity and MT.

**FIGURE 4:**
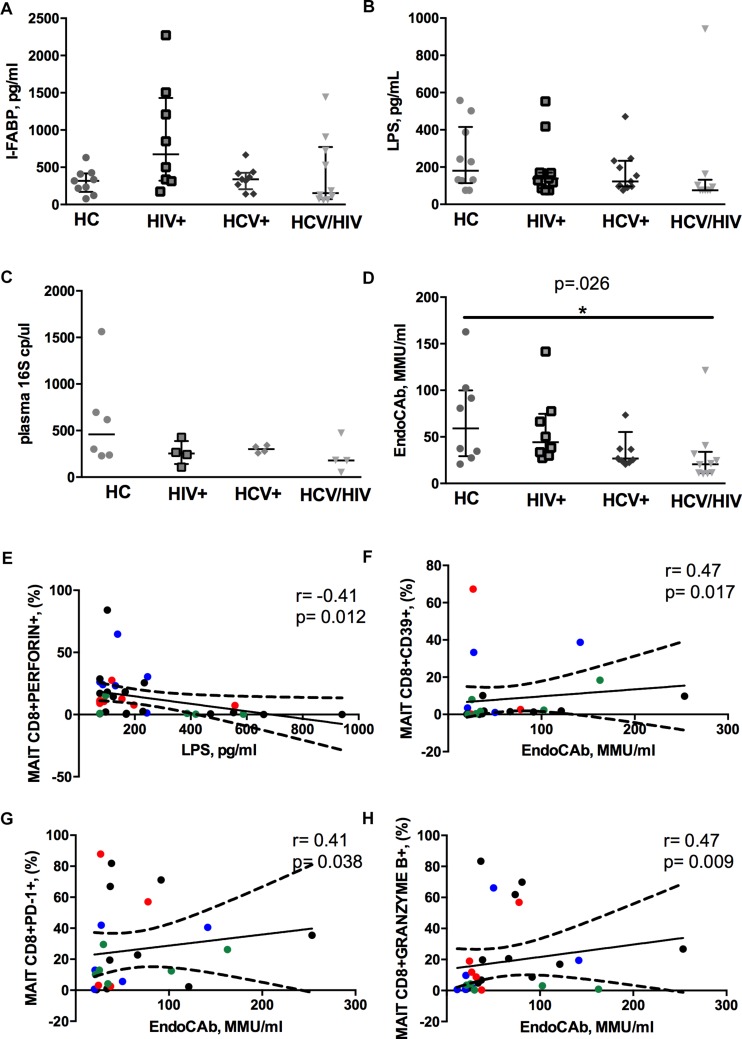
Circulating microbial translocation and gut damage markers and their associations with Vα7.2+CD161++CD8+. The lines indicate the significant comparison between two groups. ^∗^ indicates the *p*-value for each pair of groups: ^∗^ *p* < 0.05, ^∗∗^ *p* < 0.01, ^∗∗∗^ *p* < 0.001. **(A)** The levels of I-FABP, marker of enterocyte damage, were similar between the donor group and the infected patients (HC: 317 pg/ml [169–418], HIV+: 675 pg/ml [319–1431], HCV+: 339 pg/ml [206–426], HCV/HIV: 154 pg/ml [72–771], *p* = 0.158). **(B)** We observed similar plasma levels of LPS (HC: 180 pg/ml [114–416], HIV+: 138 pg/ml [86–170], HCV+ 123 pg/ml [96–234], HCV/HIV: 75 pg/ml [75–132], *p* = 0.188) between the study groups. **(C)** Circulating 16S rDNA in a subgroup of 5 patients per group was comparable (HC: 458 cp/ml [235–913], HIV+: 253 cp/ml [141–387], HCV+: 301 cp/ml [264–338], HCV/HIV: 179 cp/ml [83–400], *p* = 0.261). **(D)** The plasma levels of endotoxin core antibodies (EndoCAb) were significantly lower in virally infected subjects, particularly HCV/HIV co-infected patients (HC: 59 MMU/ml [29–100], HIV+ 44 MMU/ml [31–75], HCV+: 27 MMU/ml [23–55], HCV/HIV: 21 MMU/ml [11–34] *p* = 0.026). **(E–H)** Each patients’ cohort is marked by a different color: HC are represented in green, HIV+ in red, HCV+ in blue, HCV/HIV co-infected patients in black. **(E)** LPS plasma levels negatively correlate with perforin-expressing MAITs (*r* = –0.41, *p* = 0.012). **(F)** EndoCAb levels were positively associated with exhausted MAITs (CD39 +): *r* = 0.47, *p* = 0.017. **(G)** Vα7.2+CD161++CD8+PD1+ T-cells were positively associated with EndoCAb levels (*r* = 0.41, *p* = 0.38). **(H)** Granzyme B-expressing MAITs positively associate with plasma EndoCAb (*r* = 0.47, *p* = 0.09).

We found similar plasma levels of I-FABP, a marker of enterocyte damage, between infected patients and controls (*p* = 0.158; Figure 4A), as well as direct markers of MT (LPS, *p* = 0.188; 16S rDNA, *p* = 0.261; Figures 4B,C). Conversely, virally infected subjects displayed significantly lower endotoxin core antibodies (EndoCAb) (*p* = 0.026; Figure 4D), supporting inefficient control over translocating bacteria.

Interesting correlative patterns were evident between MT and MAIT phenotype: perforin-expressing MAITs negatively correlated with circulating LPS (*r* = –0.41, *p* = 0.012; Figure 4E), while activated/exhausted CD39+/PD-1+ and granzyme B+ MAITs positively associated with EndoCAb (CD39+: *r* = 0.47, *p* = 0.017; PD1+: *r* = 0.41, *p* = 0.038; granzyme B+: *r* = 0.47, *p* = 0.009; Figures 4F–H).

### Virally Infected Subjects Show Similar Vα7.2+CD161-CD8+, Higher Immune Activation/Exhaustion and Cytolysis

Because CD161 cell-surface down-regulation was suggested as a mechanism behind MAIT cell depletion during chronic infections (Leeansyah et al., 2013), we sought to investigate the phenotype and function of Vα7.2+CD161-CD8+ T-cells (gating strategy shown in Figure 1).

**FIGURE 5:**
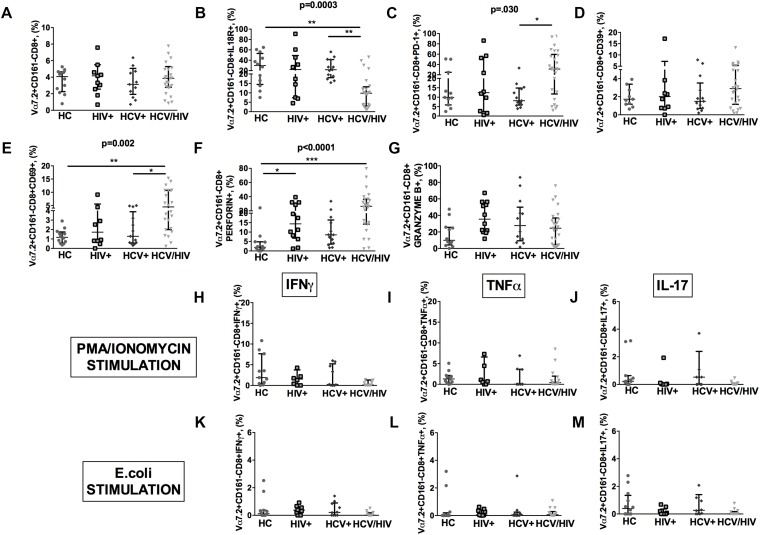
Frequency, phenotypes and cytokine production of Vα7.2+CD161-CD8+ T-cells. The lines indicate the significant comparison between two groups. ^∗^ indicates the *p*-value for each pair of groups: ^∗^ *p* < 0.05, ^∗∗^ *p* < 0.01, ^∗∗∗^ *p* < 0.001. **(A)** The frequency of circulating Vα7.2+CD161-CD8+ subset was similar among the four study groups (HC: 4.07%, HIV+: 4.17%, HCV+: 3.13%, HCV+/HIV+: 3.87%, *p* = 0.918). **(B)** HCV+/HIV+ co-infected patients featured significantly lower levels of IL18R (HC: 29.8%, HIV+: 22%, HCV+: 21.7%, HCV+/HIV+: 9.81%, *p* = 0.0003). **(C)** Similar levels of CD39 on Vα7.2+CD161-CD8+ were found (HC: 1.73%, HIV+: 2.01%, HCV+: 1.49%, HCV+/HIV+: 2.89%, *p* = 0.667). **(D)** Vα7.2+CD161-CD8+PD-1+ T-cells were significantly increased in co-infected subjects (HC: 9.89%, HIV+: 12.4%, HCV+: 8.15%, HCV+/HIV+: 31.2%, *p* = 0.030). **(E)** The highest levels of Vα7.2+CD161-CD8+CD69+ were found in HCV/HIV patients (HC: 1.17%, HIV+: 1.72%, HCV+: 1.28%, HCV+/HIV+: 4.46%, *p* = 0.002). **(F)** We observed significantly higher levels of perforin (HC: 1.91%, HIV+: 14.3%, HCV+: 8.54%, HCV+/HIV+: 26.5%, *p* < 0.0001) in virally infected patients. **(G)** Similar granzyme B levels were found in virally infected patients, as compared to the control group (HC: 10%, HIV+: 35.4%, HCV+: 27.7%, HCV+/HIV+: 24.6%, *p* = 0.105). Vα7.2+CD161-CD8+ T-cell population produced very limited cytokine amount following both **(H–J)** PMA/ionomycin and **(K–M)** *E. coli* stimulation, with no differences between the study groups.

HCV+/HIV+ co-infected patients featured similar Vα7.2+CD161-CD8+ cell proportions (*p* = 0.918; Figure 5A), significantly lower IL18R+ (*p* = 0.0003; Figure 5B), and higher PD-1+ (*p* = 0.030; Figure 5C), yet similar CD39+ (*p* = 0.667; Figure 5D) and higher CD69+Vα7.2+CD161-CD8+ (*p* = 0.002; Figure 5E). Virally infected patients displayed significantly higher levels of perforin (*p* < 0.0001; Figure 5F) and similar granzyme B+ expression in Vα7.2+CD161-CD8+ cells (*p* = 0.105; Figure 5G).

As to their cytokine-producing profile, Vα7.2+CD161-CD8+ population produced very limited quantities of cytokines following both PMA/ionomycin and *E. coli* exposure, with no differences between the study groups (Figures 5H–M).

### Gut Microbiota Correlates With Frequency and Function of Vα7.2+CD161++CD8+ and Vα7.2+CD161-CD8+

Given the role of MAIT cells in gut immunity and in anti-bacterial response (Napier et al., 2015), we next questioned whether MAIT defects in our cohort of virally infected patients might be linked to gut microbiota dysbiosis.

### Gut Microbiota Dysbiosis in Virally Infected Patients

**FIGURE 6:**
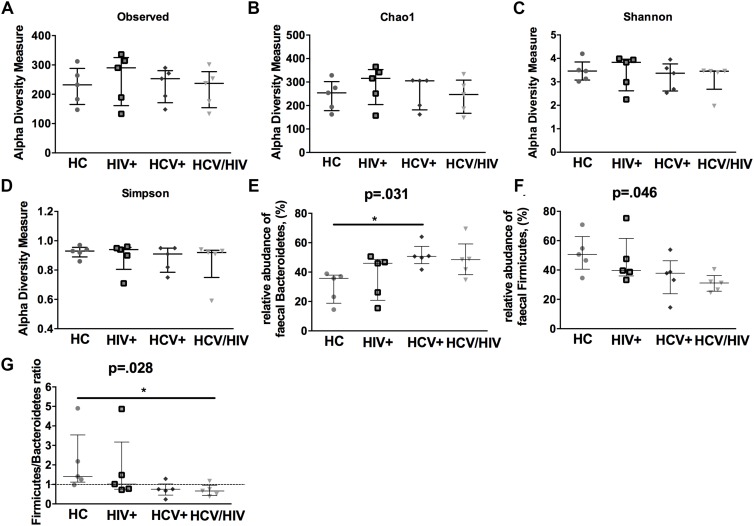
Alpha-diversity indexes and fecal bacteria relative abundance. The lines indicate the significant comparison between two groups. ^∗^ indicates the *p*-value for each pair of groups: ^∗^ *p* < 0.05, ^∗∗^ *p* < 0.01, ^∗∗∗^ *p* < 0.001. We calculated **(A,B)** richness (observed and Chao1) and evenness **(C,D)** (Shannon and Simpson) indexes, failing to find any differences among the study groups. **(E)** higher relative abundance of *Bacteroidetes* (*p* = 0.031), **(F)** lower relative abundance of *Firmicutes* **(G)** and lower *Firmicutes*/*Bacteroidetes* ratio (*p* = 0.028).

In a subgroup of 5 subjects per group, we first evaluated the gut microbiota complexity, by calculating richness (observed and Chao1) and evenness (Shannon Entropy and Simpson) indexes and failed to find any differences among the study groups (Figures 6A–D).

We further characterized the relative proportion of fecal bacteria species at each taxonomic level (phylum, class, order, family, genus and species), finding significant differences between virally infected individuals and healthy controls. Interestingly, virally infected patients and particularly co-infected individuals displayed higher relative abundance of *Bacteroidetes* (*p* = 0.031; Figure 6E), lower relative abundance of *Firmicutes* (*p* = 0.046; Figure 6F) and lower *Firmicutes*/*Bacteroidetes* ratio (*p* = 0.028; Figure 6G).

**FIGURE 7:**
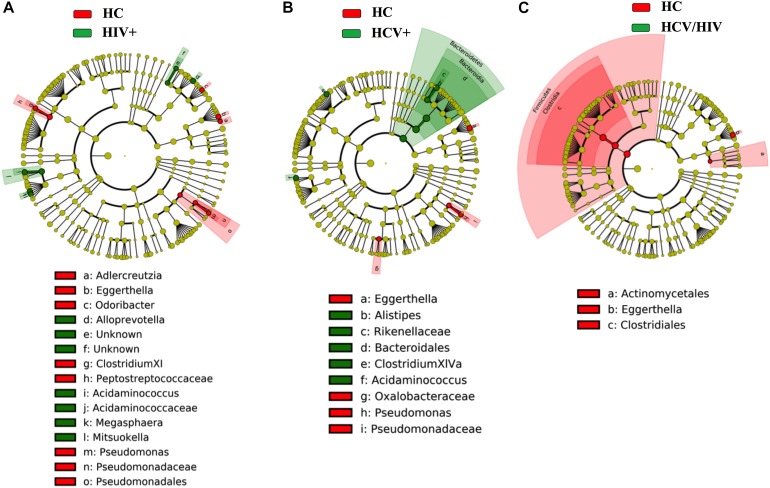
Fecal bacteria beta diversity analysis: LEfSe Analysis. Analysis of differences in the microbiota between the study groups using LEfSe software (LDA, coupled with effect size measurement). **(A–C)** HCV+ individuals (in green) featured higher content of bacteria belonging to the *Bacteroidetes* phylum, HCV+/HIV+ loss of bacteria belonging to the *Firmicutes* phylum as compared to healthy controls (in red).

Finally, by using the linear discriminant analysis (LDA) effect size (LEfSe) with LDA score >2 as the cut-off, we observed an enrichment of *Bacteroidetes* phylum in HCV+ and an impoverishment in *Firmicutes* phylum in HCV+/HIV+ when compared to HC (Figure 7), with no differences among virally infected groups (data not shown).

### Correlations Between Gut Microbiota Composition, MAIT Cells and CD161- T-Cells

We next assessed whether differences in gut microbiota composition might be connected to the disrupted MAIT cell and Vα7.2+CD161- T-cell frequency and function.

**FIGURE 8:**
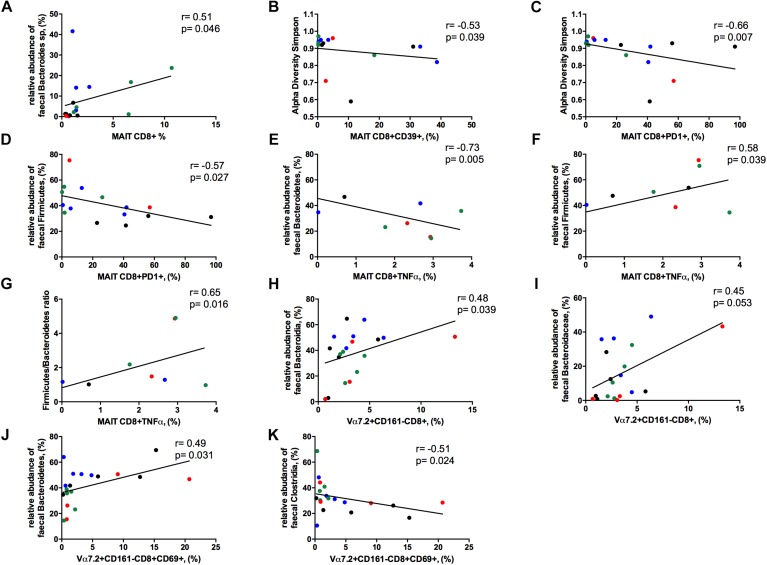
Correlations between Vα7.2+CD161++CD8+ or Vα7.2+CD161-CD8+ and gut dysbiosis. Each patients’ cohort is marked by a different color: HC are represented in green, HIV+ in red, HCV+ in blue, HCV/HIV co-infected patients in black. **(A)** The frequency of circulating MAITs was positively associated with the relative abundance of *Bacteroides* spp. (*r* = 0.51, *p* = 0.046). **(B)** The levels of exhausted Vα7.2+CD161++CD8+39+ were negatively correlated with alpha diversity Simpson index (*r* = –0.53, *p* = 0.039). **(C)** Vα7.2+CD161++CD8+PD1+ negatively associated with alpha diversity Simpson index (*r* = –0.66, *p* = 0.007). **(D)** PD-1-expressing MAITs showed an inverse association with the relative abundance of *Firmicutes* (*r* = –0.57, *p* = 0.027). MAIT ability to produce TNFα following bacterial challenge was negatively associated with the **(E)** relative abundance of *Bacteroidetes* (*r* = –0.73, *p* = 0.005) and positively with the **(F)** relative abundance of *Firmicutes* (*r* = 0.58, *p* = 0.039) and **(G)** with *Firmicutes/Bacteroidetes* ratio (*r* = 0.65, *p* = 0.016). **(H)** The frequency of Vα7.2+CD161-CD8+ T-cells were positively associated with the order *Bacteroidia* (*r* = 0.477, *p* = 0.039). **(I)** The family of *Bacteroidaceae* correlates with the proportion of Vα7.2+CD161-CD8+ T-cell subset (*r* = 0.45, *p* = 0.053). **(J)** The activated Vα7.2+CD161-CD8+ were positively associated to the relative abundance of *Bacteroidetes* (*r* = 0.49, *p* = 0.031) **(K)** CD69-expressing Vα7.2+CD161-CD8+ T-cells negatively correlated with the order *Clostridia* (*r* = –0.51, *p* = 0.024).

The frequency of circulating MAIT cells was positively associated with the relative abundance of *Bacteroides* spp. (*r* = 0.51, *p* = 0.046; Figure 8A), some of which are known to be inducers of regulatory T-cell functions (Troy and Kasper, 2010).

The levels of activated/exhausted CD39+ and PD1+ MAIT cells correlated negatively with alpha diversity Simpson index (*r* = –0.53. *p* = 0.039; *r* = –0.66. *p* = 0.007, respectively; Figures 8B,C). Moreover, PD-1-expressing MAIT showed an inverse association with the relative abundance of *Firmicutes* (*r* = –0.57, *p* = 0.027; Figure 8D). Interestingly, MAIT ability to produce TNFα following bacterial challenge negatively associated with the relative abundance of *Bacteroidetes* (*r* = –0.73, *p* = 0.005; Figure 8E) and positively with the relative abundance of *Firmicutes* (*r* = 0.58, *p* = 0.039; Figure 8F) and with *Firmicutes/Bacteroidetes* ratio (*r* = 0.65, *p* = 0.016; Figure 8G).

Contrarily, Vα7.2+CD161-CD8+ T-cells positively associated with the order *Bacteroidia* (*r* = 0.48, *p* = 0.039; Figure 8H) with a trend toward the family of *Bacteroidaceae* (*r* = 0.45, *p* = 0.053; Figure 8I). Besides, the levels of activation on this subset were positively associated to the relative abundance of *Bacteroidetes* (*r* = 0.49, *p* = 0.031; Figure 8J) and negatively correlated with the order *Clostridia* (*r* = –0.51, *p* = 0.024; Figure 8K).

We found no association between MAIT cells and markers of HIV disease progression (CD4, HIV-RNA levels, duration of infection and of cART), and HCV disease progression (HCV-RNA levels and length of infection).

## Discussion

In chronically HIV- and/or HCV-infected patients, we hereby show a significant loss of circulating MAIT cells, the residual proportion of which display higher production of perforin and granzyme B, and yet an impaired *ex vivo* function. Further, Vα7.2+CD161-CD8+ T-cells, despite equally frequent in virally infected and healthy individuals, feature an activated phenotype and yet appear functionally impaired. Interestingly, both MAIT cells and Vα7.2+CD161-CD8+ T-cells significantly associate with gut dysbiosis.

In line with literature data (Barathan et al., 2016; Spaan et al., 2016), our study shows a massive depletion of the MAIT compartment in both HIV- and HCV-infected populations, with no differences among mono- and co-infection. The residual MAIT cells are functionally exhausted and display higher cytolysis markers, possibly indicating continuous antigen exposure. Given that microbial translocation may stimulate innate immune cells via TLR pathways, leading to systemic immune activation (Ussher et al., 2016), we explored the association between activated/exhausted MAIT phenotypes and microbial translocation. Interestingly, while LPS plasma levels negatively correlate with perforin-expressing MAIT cells, supporting the role of endotoxin in hampering MAIT function, EndoCAb levels were positively associated with exhausted/activated (CD39/PD1) and granzyme B-expressing MAIT cells, suggesting the effort of the immune system to fight continuous microbial threat. Some studies suggested that rather than being depleted, MAIT cells have an altered phenotype, namely, the down-regulation of CD161; Leeansyah et al. (2013) suggested that continuous exposure to bacterial products in HIV disease may lead to MAIT cell exhaustion and loss, with concomitant expansion of the CD161neg population originated from the MAIT cells. Similarly, Freeman et al. (2017) showed that in immune failure patients, the reduction in peripheral MAIT cells seems to be due, at least in part, to a loss in CD161 expression, and not merely the result of trafficking into mucosal tissues or cell death. By contrast other studies, argued that Vα7.2+CD161-CD8+ are not MAIT cells (Leeansyah et al., 2013; Sandberg et al., 2013; Fergusson et al., 2014), since they do not retain their functions. In our cohort, we describe a Vα7.2+CD161-CD8+ T cell phenotype/function divergent from MAIT T cells, with preserved frequency, inefficient cytokine production, and no association with microbial translocation, altogether supporting the idea of a distinct T-cell population. However, given that the MAIT cell loss is a continuous long-term process, we could not exclude that what we described is the result of different and concomitant mechanisms, such as homing to sites of inflammation, exhaustion, and also loss of CD161 expression late in chronic diseases.

Having shown altered MAIT phenotypes during chronic viral infections, we next asked whether their anti-bacterial properties were also impaired. Interestingly, compared to healthy controls, we found that the *ex vivo* reactivity of MAIT cells to cells cultured together with bacteria is very weak, with HCV+/HIV+ co-infected patients displaying the lowest cytokine production, implying a defect in MAIT antimicrobial functions, possibly due to tolerance driven by continuous microbial translocation. However, the lack of association between MAIT ability to face bacteria challenge and MT markers, seems to suggest that other mechanisms might be involved, such as continuous MAIT engagement, in turn resulting in functional exhaustion, defects in the triggering and/or in signal transduction or deficiency in cytokine production/release.

Given the lack of direct MAIT stimulation by viruses, our finding of such MAIT impairment in both HIV and HCV chronic infections, raises the question of whether other factors rather than the viruses themselves are involved (van Wilgenburg et al., 2016). In the last few years, both HIV- and HCV-infected patients have been proven to feature a marked dysbiosis, that is not reverted by antiviral treatments (Bajaj et al., 2016; Williams et al., 2016). The homeostasis between microbiota, intestinal epithelium and innate and adaptive immunity favors the predominance of regulatory networks that prevent inflammation or immune-mediated disease (Duerkop et al., 2009; Maynard et al., 2012; Powell and MacDonald, 2017). Recently, some authors have reported a direct link between MAIT cells and gut microbiota. Indeed, MAIT cells have been demonstrated to discriminate and categorize complex human microbiota through computation of TCR signals and to shape their phenotypes and responses according to local environment-driven microbial metabolism (Schmaler et al., 2018; Tastan et al., 2018). Thus, we hypothesized that MAIT disruption might be associated with a decreased frequency of bacteria involved in epithelial barrier health and immune-regulation, and increased abundance of bacteria with known pro-inflammatory potential in the setting of chronic viral infections.

To test this hypothesis, we first investigated gut microbiota composition, confirming a profound dysbiosis in virally infected patients, mainly in HCV+/HIV+ co-infected individuals, delineating a *Firmicutes*-poor/*Bacteroidetes-*rich microbiota. Indeed, the *Firmicutes phylum* comprises some commensal bacteria with immune regulatory properties, while by contrast the *Bacteroidetes phylum* includes bacteria with proinflammatory properties (Eckburg et al., 2005). In view of this, our finding of a shift toward lower *Firmicutes*, such as *Clostridia*, might support the role of gut dysbiosis in sustaining immune activation as a consequence of regulatory function loss, possibly leading to clinical progression. The factors associated with HIV and/or HCV-driven dysbiosis still remain to be fully elucidated, whether it is elicited by the viruses themselves, or rather it is a consequence of differences in lifestyle (i.e., MSM vs. heterosexual), co-morbidities and the use of chronic therapies. Interestingly, our results seem to closely link gut dysbiosis and MAIT compartment. Indeed, we found a positive association between MAIT frequency and *Bacteroides* spp., some of which are reported to be inducers of regulatory T-cell function (Troy and Kasper, 2010), leading us to hypothesize a role of certain bacteria in modulating MAIT cells, probably improving the anti-bacterial properties of the immune system. A reduced diversity of the gut microbiota has been associated with immune dysfunction and reduced CD4 T-cell counts (Nowak et al., 2015), our observation of an inverse correlation between exhausted MAIT and alpha diversity suggests an important role of low microbiota diversity in weakening the efficiency of the host immune system.

In addition, we have shown an association between TNFα-producing MAIT cells and gut microbiota composition, with bacteria belonging to the *Firmicutes phylum* positively correlating with MAIT TNFα production following *ex vivo E. coli* stimulation, while bacteria belonging to the *Bacteroidetes phylum* negatively associating with MAIT TNFα release. Despite the fact that the small number of samples tested might limit the statistical power of the findings, our data altogether seem to suggest a link between gut dysbiosis and inflammation, supporting the hypothesis of a direct effect of altered microbiota composition on immune function, possibly posing viral-mediated dysbiosis as a central player in the hampered anti-bacterial MAIT capability. As intriguing and interesting as it is, we acknowledge that the few samples tested do not allow definitive conclusions to be drawn and need to be confirmed in larger cohorts.

Lastly, we didn’t find any association between MAIT cells and usual markers of both HIV and HCV diseases, probably as a reflection of the long history of infection, hence supporting the theory of significant and permanent damages of MAIT compartment early in the infection.

To our knowledge this is the first study to investigate the associations between gut microbiota and MAIT cells in chronic HIV and HCV infections. The correlative nature of our study did not allow us to show a causal relationship, however, the observations that certain bacteria may associate to MAIT activation/function support the hypothesis of a reciprocal interaction of MAIT cells and intestinal microbiota, paving the way to possible future research on microbiome-targeted therapy aimed at restoring mucosal immunity in chronic HIV and HCV infections. The next step, i.e., the *in vitro* stimulations of the MAIT cells using representative members of the identified phyla/genera will clarify the mechanisms and the effects exerted by these bacteria on MAIT cell population.

Several limitations must be acknowledged. The small sample size might have hampered the ability to detect a relationship between MAIT cells and liver fibrosis that has been described in literature (Beudeker et al., 2018; Hegde et al., 2018). Likewise, microbiota analyses on larger cohorts with extensive evaluation of microbial community function, might have provided broader pathogenic insight. Similarly, the study of bacteria composition, gut damage and MAIT cells directly at mucosal sites, might have shed light onto the causal relationship between mucosal adherent microbiota. Furthermore, given the limited sample size we were forced to merge the patient groups in the correlative analyses. Thus, we could not exclude that by mixing cohorts of patients with markedly different viruses we might have lost some differences. Finally, the availability of CD4 count in HIV non-infected groups might have helped us in explaining some of our finding.

In conclusion, the MAIT compartment is profoundly impaired in chronic HIV and HCV, possibly as a consequence of the marked dysbiosis that features both infections. The microbial and environmental signals that lead to the migration, differentiation, expansion and maintenance of unconventional T-cells under physiological and pathological conditions are poorly defined and need to be further studied. An additional imperative is to explore whether differences in microbial communities result into modification in key bacterial functional pathways that drive mucosal immune dysfunction.

## Data Availability

All datasets generated for this study are included in the manuscript and the Supplementary Files.

## Ethics Statement

This study was carried out in accordance with the recommendations of “Comitato Etico, ASST Santi Paolo e Carlo” with written informed consent from all subjects. All subjects gave written informed consent in accordance with the Declaration of Helsinki. The protocol was approved by the “Comitato Etico, ASST Santi Paolo e Carlo.”

## Author Contributions

EM and MC designed the study, designed and performed experiments, analyzed and interpreted the data, designed the figures, and wrote the manuscript. BvW, LS, and EC performed the experiments and helped with analyzing the data. AdM helped with interpreting the results and edited the manuscript. PK and GM conceived and designed the study, interpreted the data, and wrote the manuscript.

## Conflict of Interest Statement

MC was awarded a European Union Lifelong Learning Program LLP/Erasmus studentship to conduct part of the analysis at the University of Oxford. GM received research grant by the Italian Ministry of Health. The remaining authors declare that the research was conducted in the absence of any commercial or financial relationships that could be construed as a potential conflict of interest.

## Supplementary Material

FIGURE S1 Frequency, phenotypes and cytokine production of Vα7.2+CD161++CD3+ T-cells. The lines indicate the significant comparison between two groups. ^∗^ indicates the *p*-value for each pair of groups: ^∗^ *p* < 0.05, ^∗∗^ *p* < 0.01, ^∗∗∗^ *p* < 0.001. **(a)** Virally infected patients were confirmed to display lower Vα7.2+CD161++CD3+ (*p* = 0.003) frequency as compared to healthy controls. **(b–e)** Similar proportion of activated/exhausted MAIT cells was observed among the study groups. **(f)** Comparable levels of perforin-producing MAIT cells. **(g)** Higher granzyme B-producing MAIT cells (*p* = 0.007) in virally infected patients as compared to healthy controls. **(h–j)** Upon PMA/ionomycin stimulation, total CD3+ MAIT cells of virally infected subjects showed lower production of cytokines (IFNγ: *p* = 0.078; TNFα: *p* = 0.030). **(k–m)** *E. coli* exposure resulted in lower cytokine production (IFNγ: *p* = 0.028; TNFα: *p* = 0.048) as compared to healthy individuals.
