# Supplementary figures and images for: Association Between Impaired Vα7.2+CD161++CD8+ (MAIT) and Vα7.2+CD161-CD8+ T-Cell Populations and Gut Dysbiosis in Chronically HIV- and/or HCV-Infected Patients
Source: Front Microbiol. 2019 Aug 28;10:1972. doi: 10.3389/fmicb.2019.01972 (PMC6722213; doi:10.3389/fmicb.2019.01972)

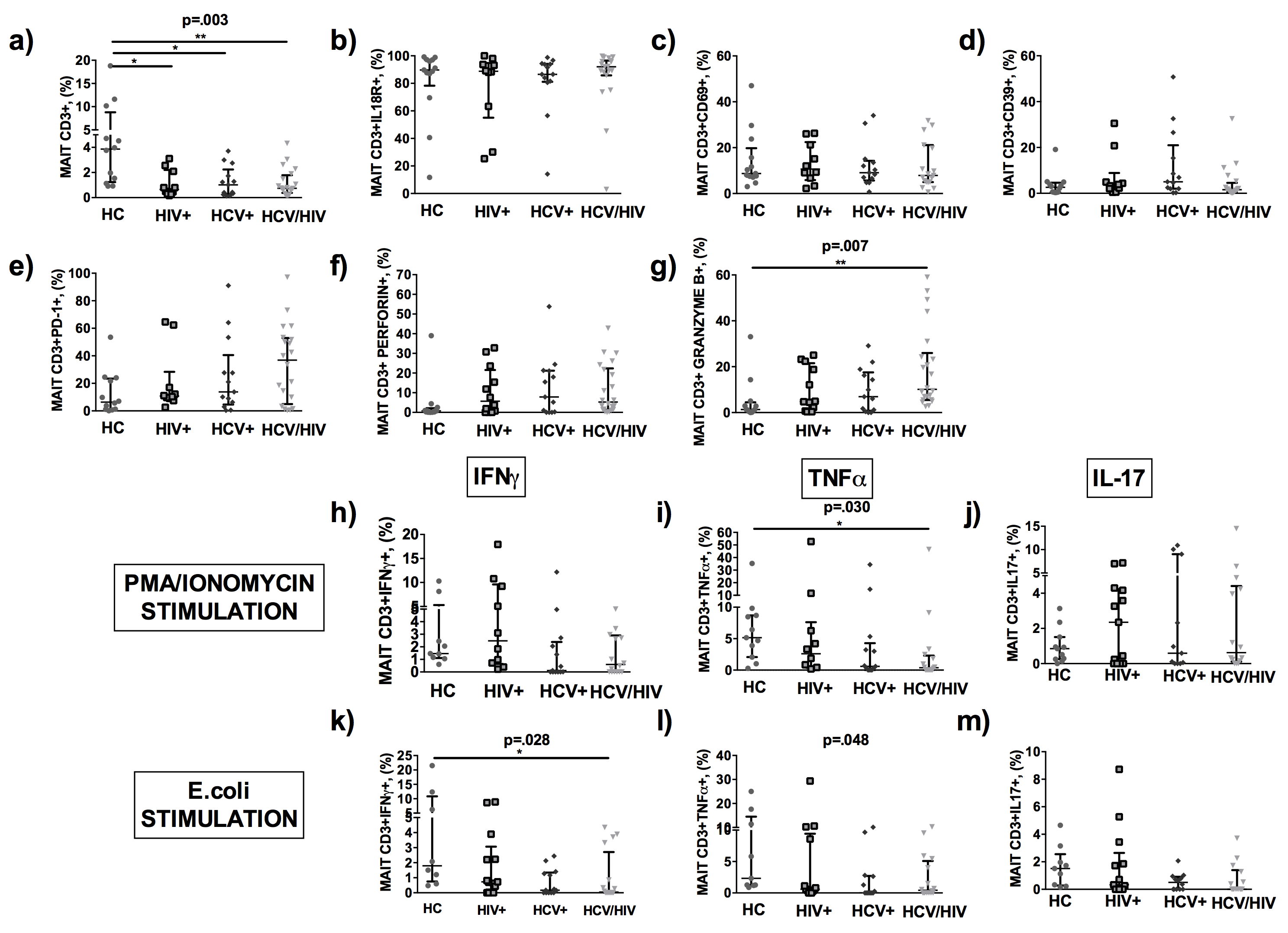

Supplement: FIGURE S1 — Frequency, phenotypes and cytokine production of Vα7.2+CD161++CD3+ T-cells. The lines indicate the significant comparison between two groups. ∗ indicates the p-value for each pair of groups: ∗ p < 0.05, ∗∗ p < 0.01, ∗∗∗ p < 0.001. (a) Virally infected patients were confirmed to display lower Vα7.2+CD161++CD3+ (p = 0.003) frequency as compared to healthy controls. (b–e) Similar proportion of activated/exhausted MAIT cells was observed among the study groups. (f) Comparable levels of perforin-producing MAIT cells. (g) Higher granzyme B-producing MAIT cells (p = 0.007) in virally infected patients as compared to healthy controls. (h–j) Upon PMA/ionomycin stimulation, total CD3+ MAIT cells of virally infected subjects showed lower production of cytokines (IFNγ: p = 0.078; TNFα: p = 0.030). (k–m) E. coli exposure resulted in lower cytokine production (IFNγ: p = 0.028; TNFα: p = 0.048) as compared to healthy individuals. [file Image_1.tiff]
